# Retraction: Dual‐Ion Stabilized Layered Structure of O–V–OforZero‐Strain Potassium Insertion and Extraction

**DOI:** 10.1002/advs.202302382

**Published:** 2023-05-26

**Authors:** Jianyi Wang, Menghui Chen, Zhida Chen, Zicong Lu, Liping Si

## Abstract

10.1002/advs.202202550

*Adv. Sci*.**2022**, *9*, 2202550

The above article from *Advanced Science*, published online on 5 June 2022 in Wiley Online Library (https://onlinelibrary.wiley.com/doi/full/10.1002/advs.202202550), has been retracted by agreement between the authors, the Editor‐in‐Chief, Kirsten Severing, and Wiley‐VCH GmbH. The retraction has been agreed as the article is based on research results and data that the authors were not authorized to use. Moreover, the majority of co‐authors have been listed despite insufficient qualification for contributorship.

10.1002/advs.202202550

*Adv. Sci*.**2022**, *9*, 2202550

The above article from *Advanced Science*, published online on 5 June 2022 in Wiley Online Library (https://onlinelibrary.wiley.com/doi/full/10.1002/advs.202202550), has been retracted by agreement between the authors, the Editor‐in‐Chief, Kirsten Severing, and Wiley‐VCH GmbH. The retraction has been agreed as the article is based on research results and data that the authors were not authorized to use. Moreover, the majority of co‐authors have been listed despite insufficient qualification for contributorship.

